# Stress granules and *Plasmodium* liver stage infection

**DOI:** 10.1242/bio.20136833

**Published:** 2013-12-18

**Authors:** Kirsten K. Hanson, Gunnar R. Mair

**Affiliations:** Instituto de Medicina Molecular, Avenida Professor Egas Moniz, Lisbon 1649-028, Portugal

**Keywords:** Host–pathogen interactions, Stress granules, *Plasmodium*, Malaria, Translational repression

## Abstract

Organisms have evolved numerous strategies to control infection by an array of intracellular pathogens. One cell autonomous pathogen control strategy is global inhibition of protein synthesis via stress granule (SG) formation. SGs are induced by stressful stimuli such as oxidative stress and nutrient deprivation, and are known to counteract both viral and bacterial infections. Pathogens, in turn, may actively block an infected cell's ability to form SGs. *In vitro* and *in vivo*, many liver stage malaria parasites are eliminated during development. We show here that SG formation is not amongst the strategies used for elimination of parasites from hepatocytes. Neither cell traversal, sporozoite invasion, nor rapid parasite growth leads to the formation of SGs. Furthermore, *Plasmodium berghei* infection does not compromise the ability of infected cells to assemble SGs in response to oxidative or nutritional stress. *Plasmodium* infection is therefore not detected by hepatocytes as a strong stressor necessitating global translational repression in response, highlighting the idea that *Plasmodium* has evolved strategies to ensure its remarkable growth in the hepatocyte while maintaining host cell homeostasis.

## Introduction

*Plasmodium* parasites are Apicomplexan protozoans with a complex lifecycle encompassing motile, non-replicative and sessile, replicative forms in both the mosquito and mammalian host. During the initial stage of infection in the mammalian host, *Plasmodium* sporozoites are transferred from an infected mosquito into the dermis. Many of these motile sporozoites then enter the circulatory system, and after arresting in the liver sinusoids, traverse several cells, before invading a final hepatocyte, and there establishing residence inside a parasitophorous vacuole. Inside this vacuole, the parasite undergoes a remarkable expansion, generating thousands of progeny in as little as two days during this clinically silent phase of infection. Once mature, these progeny, called merozoites, will initiate the next phase of the *Plasmodium* life cycle, during which continuous cycles of red blood cell invasion, parasite replication, and red blood cells lysis will give rise to the symptoms and syndromes of malaria.

Not all growing exoerythrocytic forms (EEFs) will successfully complete development, though. Different mouse strains show different susceptibilities to infection initiated by *P. berghei* sporozoites (Khan and Vanderberg, 1991) despite initially similar liver parasite loads (Gonçalves et al., 2008). *In vitro*, a similar phenomenon has been documented, with EEF numbers decreasing over the natural time-course of infection (Leiriao et al., 2005). The true endpoint of the liver stage of infection is the release of hepatic merozoite-filled merosomes into the sinusoidal bloodstream of the host (Sturm et al., 2006), a process that also occurs in HepG2 cells *in vitro*. A recent report suggests that only 20–30% of infected HepG2 cells detach, indicating that the majority of parasites cells do not reach maturity (Falkard et al., 2013). While the innate immune system in a naïve host certainly plays a role in controlling infection *in vivo* (Liehl and Mota, 2012), EEF attrition *in vitro* can only be hepatocyte-mediated, or due to intrinsic failure in parasite development. The relative contributions of host-cell-mediated anti-*Plasmodium* defense strategies and developmental failure in the EEF, and the mechanisms behind both, remain to be discovered. Whether or not the host cell can specifically recognize the parasite via pathogen-associated molecular patterns (PAMPs) (Liehl and Mota, 2012), the infection process could induce cellular stress responses in the host cell during the invasion process itself, which ends in the internalization of a sizeable (roughly 10 µm long, 2 µm in diameter) object into the cell cytoplasm, or during the remarkable EEF growth that occurs after the onset of DNA replication (Prudêncio et al., 2006).

Eukaryotic cells have evolved numerous ways to cope with intra- and extracellular stressors. One important mechanism to overcome unfavorable growth conditions lies in transient inhibition of protein translation mediated through phosphorylation of key factors such as eIF2α by specific kinases. As a result, ribosome-bound transcripts are translationally silenced and sequestered in localized, cytoplasmic foci of the cell, so-called stress granules (SG); they contain stalled pre-initiation complexes, non-translating mRNA and characteristically incorporate Ras-GAP SH3-domain binding protein 1 (G3BP) and T-cell-restricted intracellular antigen 1 (TIA-1) (Anderson and Kedersha, 2008).

Downregulating protein translation while maintaining mRNAs in such quiescent mRNPs allows the cell to quickly resume protein translation once the stress has been alleviated (Brengues et al., 2005). SGs form during oxidative stress, UV exposure, heat shock and lack of nutrients (glucose starvation for example) in yeast and mammalian cells (Anderson and Kedersha, 2008; Kedersha and Anderson, 2007; Nissan and Parker, 2008), and are assembled during infection with a wide range of viruses (Beckham and Parker, 2008; White and Lloyd, 2012). SG formation may have evolved as an effective cell-autonomous strategy employed to fight viral infections, which rely on the translational machinery of infected cells for viral protein production; SG formation can thus block viral replication (Beckham and Parker, 2008). However, viruses including poliovirus have evolved counterstrategies: they actively block the host cell's ability to form SGs during the infection (Lloyd, 2012), allowing translation of viral RNAs to continue, and so thwarting an otherwise powerful defence mechanism that may form part of the innate immune system. The bacterium *Shigella* has been shown to cause SG formation in infected cells, albeit as a response to pathogen-induced amino acid starvation (Tattoli et al., 2012). As in *Salmonella*, *Shigella* can also cause the disassembly of P bodies (Eulalio et al., 2011), which function in mRNA decay, translational repression and Argonaute-mediated gene silencing, and interact with, and receive mRNAs targeted for degradation from SGs. Cellular SG formation capability is typically measured by cellular SG formation in response to oxidative stress from arsenite treatment, which is the most accepted standard for canonical SG formation; G3BP is a robust SG marker that is routinely employed to visualize cellular SG foci, induced by a range of stressors (Kedersha and Anderson, 2007).

During *Plasmodium* liver stage infection, inhibition of the host cell protein translation machinery via SG formation could occur as a protective response to the stress of cell traversal and invasion by sporozoites, or due to EEF growth. Host cell SG formation could deprive the parasite of host resources, ultimately contributing to its elimination. Conversely, *Plasmodium* parasites could actively subvert the host cell's ability to respond to cellular stress by SG formation. Here we address for the first time both of these questions: whether *Plasmodium* liver stage infection induces SG formation, and how an infected cell's ability to respond to oxidative or nutritional stressors may be altered during the course of EEF development.

## Results and Discussion

**Fig. 1.**
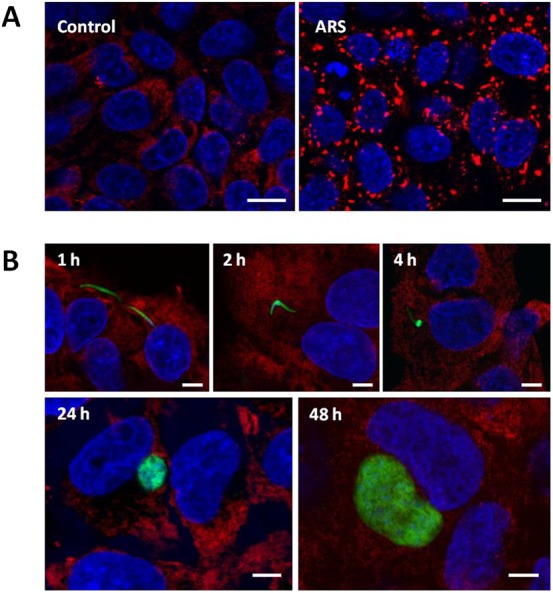
*Plasmodium berghei* infection does not induce G3BP+ stress granule formation in HepG2 hepatocytes. (A) G3BP-defined SG form in HepG2 cells following arsenite treatment. Diffuse G3BP-staining (red) in untreated cells (left panel) accumulates into clearly visible SG foci (right panel) following arsenite addition. Nuclei are stained with DAPI (blue). Scale bars: 10 µm. (B) A mixture of infected and non-infected HepG2 cells are shown at indicated time-points (hours) following addition of GFP+ *P. berghei* sporozoites (green) and detection of the SG marker G3BP (red). Nuclei were stained with DAPI (blue). Scale bars: 5 µm.

Hepatoma cell lines and the rodent malaria parasite are routinely used in the study of host–parasite interactions during liver stage of *Plasmodium* infection. Here we studied the effect of *P. berghei* infection on SG formation in HepG2 cells. As expected, non-infected HepG2 cells cultured in standard conditions uniformly lacked evidence of SG formation, as assayed by G3BP-staining of paraformaldehyde-fixed cells. G3BP was dispersed throughout the cytoplasm in these cells (Fig. 1A). A 45 minute incubation with 1 mM Sodium (meta)arsenite (arsenite) was sufficient to induce SG formation in all non-mitotic HepG2 cells (Fig. 1A). Mitotic cells are known to be incapable of SG formation (Sivan et al., 2007), but all other cells responded with development of prominent G3BP-positive foci.

**Fig. 2.**
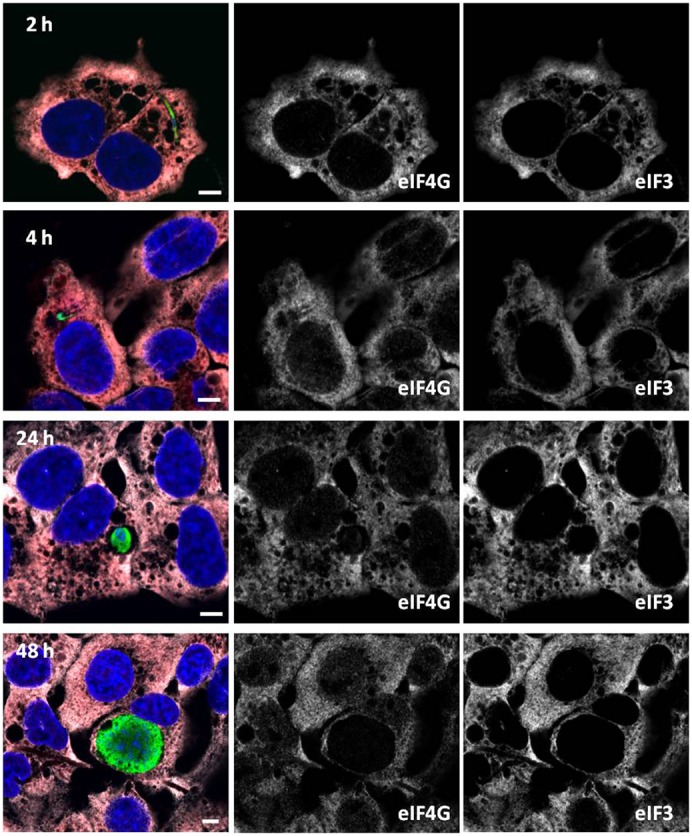
eIF4G and eIF3η are not found in stress granules in *Plasmodium*-infected cells. A mixture of infected and non-infected HepG2 cells are shown at indicated time-points (hours) following addition of *P. berghei* sporozoites. Representative images with *Plasmodium* parasites are shown in green (anti-HSP70), and detection of the SG markers eIF4G (red in merged image) and eIF3η (white in merged image). Nuclei were stained with DAPI (blue). Individual grayscale images of the largely overlapping eIF4G and eIF3η as labeled. Scale bars: 5 µm.

We next tested whether liver stage *Plasmodium* infection would be capable of inducing SG formation in cell culture. After infection of HepG2 cells with *P. berghei* salivary gland sporozoites, we monitored SG formation at distinct time-points during parasite development to assess whether the sporozoite migration and invasion processes, or EEF development infection would induce SG formation. At 1 and 2 hours post-sporozoite addition (p.i.) all cells (both those containing a parasite and those without) were uniformly SG-negative (Fig. 1B), indicating that neither cell traversal nor invasion by the parasite induces SG formation. Furthermore, all host cells remained G3BP-SGnegative throughout hepatic trophozoite and schizont development, as assayed at 4, 24, and 48 hours p.i. (Fig. 1B). We confirmed these findings using two additional SG markers, eukaryotic initiation factor (eIF) 4G and eIF3η (Kedersha and Anderson, 2007). Both eIF4G and eIF3η remain distributed throughout the cytoplasm in *Plasmodium-*infected cells at 2, 4, 24 and 48 h post-infection (Fig. 2), with no sign of SG formation in any infected cell. These observations clearly show that no step of the liver stage *Plasmodium* infection through to parasite schizogony leads to global inhibition of host cell protein translation through SG formation.

**Fig. 3.**
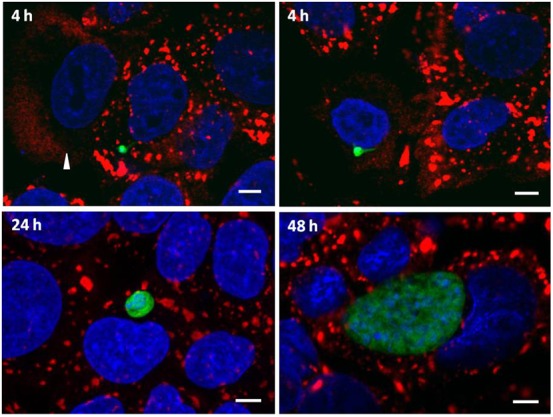
Stress granule formation is largely unaffected in *Plasmodium berghei* infected HepG2 cells. *Plasmodium*-infected cells respond to arsenite exposure with G3BP+ SG formation throughout the course of liver stage infection; representative images shown for 4 h (top left), 24 h (bottom left) and 48 h (bottom right). Exclusively at the 4 h timepoint, rare SG-infected cells could be found (top right), but similarly SG-non-infected cells (arrowhead; top left panel) were also found. G3BP (red), *P. berghei*-GFP (green), DAPI (blue). Scale bars: 5 µm.

**Fig. 4.**
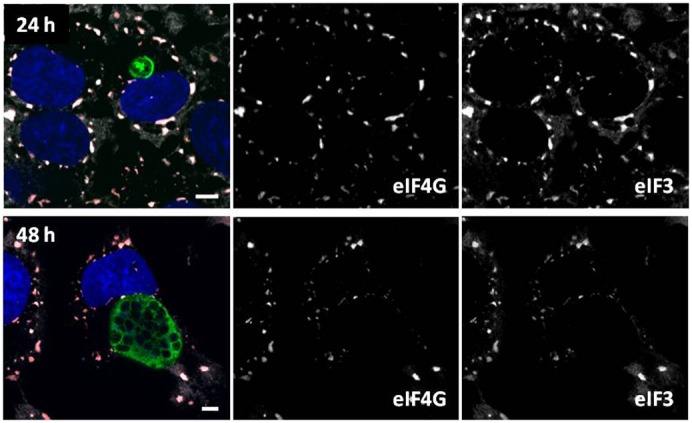
eIF4G and eIF3η localize to stress granules after oxidative stress regardless of *P. berghei* infection status. *Plasmodium*-infected cells, as well as non-infected cells, respond to arsenite exposure with SG formation throughout the course of liver stage infection; representative images shown for 24 h and 48 h. *P. berghei* EEFs are labeled with anti-HSP70 (green), along with the SG markers eIF4G (red in merged image) and eIF3η (white in merged image). Nuclei were stained with DAPI (blue). Individual grayscale images of the largely overlapping eIF4G and eIF3η as labeled. Scale bars: 5 µm.

We next tested whether cells infected by the malaria parasite remained capable of SG formation in response to oxidative stress, as cells infected with other pathogens lose this ability over time (Eulalio et al., 2011; Panas et al., 2012; Tattoli et al., 2012; White et al., 2007). We subjected coverslips containing infected cells to arsenite treatment at 4, 24 and 48 hours post-infection. At each time-point, G3BP-positive SGs were clearly induced in both infected and non-infected cells (Fig. 3). At the 24 and 48 h timepoints 100% of infected cells had SG after a 45 minute arsenite treatment (Table 1), while at 4 hours post-infection, there were rare cells, both infected and non-infected, in which SGs did not form (Fig. 3). As cell traversal by *Plasmodium* sporozoites is known to result in some cell death (Frevert et al., 2005), we speculate that these may be pre-morbid cells that have failed to recover from the process of traversal. We further confirmed that eIF4G and eIF3η are found in SG in both non-infected and infected cells subjected to arsenite treatment at 24 h and 48 h post-infection (Fig. 4).

**Table 1.**
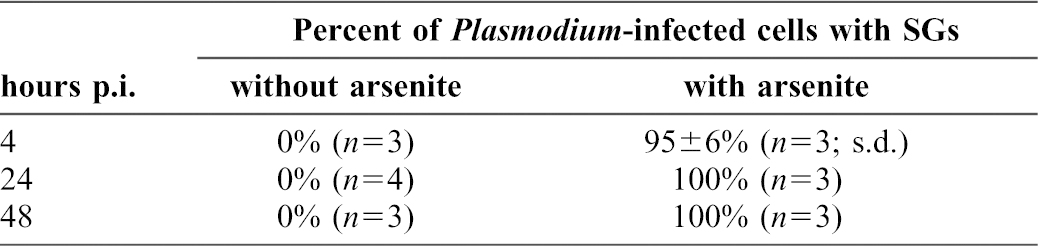
Quantification of infection-induced and arsenite-induced stress granules in control and *P. berghei*-infected HepG2 cells.

**Fig. 5.**
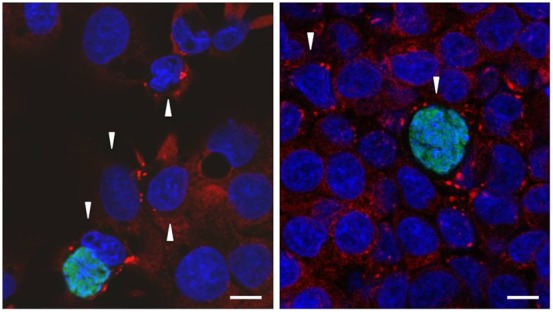
Nutrient starvation induces stress granule formation in infected and uninfected HepG2 cells alike. Representative images showing G3BP+ SG in both infected and non-infected HepG2 cells 55 h post-sporozoite addition, after 3 h of serum and glucose deprivation. Arrowheads indicate cells (both infected and non-infected) that contain SGs.G3BP (red), *P. berghei*-GFP (green), DAPI (blue). Scale bars: 5 µm.

While oxidative stress is perhaps the most potent inducer of SGs and the most often studied, we investigated whether HepG2 cells could similarly employ a global translational shutdown in response to essential nutrient deprivation. Nutrient deprivation has long been known to cause SG formation, which can be induced by experimental glucose starvation (Kedersha and Anderson, 2007), but also by amino acid deprivation due to *Shigella* infection (Tattoli et al., 2012). *Plasmodium* liver stages are believed to rely, in part, on the host cell milieu for essential nutrients. Lipids (Bano et al., 2007), fatty acids (Favretto et al., 2013; Mikolajczak et al., 2007), along with other host resources must be scavenged due to *Plasmodium* metabolic limitations (Ginsburg, 2009) during liver stage development. We tested whether SGs could be induced in HepG2 cells in response to a 3-hour nutrient depletion. HepG2 cells grown in glucose-free MEM (with serum) for 3 hours did not present any SGs (not shown), while 3 hours in serum- and glucose-free medium was capable of inducing SGs in HepG2 cells (Fig. 5). Many fewer cells exhibited SGs after the 3-hour serum and glucose starvation period, as compared with a brief arsenite treatment in which nearly 100% showed SGs. Importantly though, starvation-induced SGs could also be observed in cells harboring mature *P. berghei* EEFs. This indicates that infected cells remain competent to sense and respond to severe nutritional stress, and that the growing EEF does not place such a drain on cellular resources.

In the mouse, SG formation involves 28 genes (GO:0010494 cytoplasmic stress granules): Atxn2, Caprin1, Cirbp, Ddx1, Ddx3x, Ddx6, Eif2s1, Eif4e, Fmr1, G3bp1, Grb7, Igf2bp1, Khsrp, Lin28a, Lsm14a, Mbnl1, Nanos3, Pabpc1, Pabpc4, Pqbp1, Pum2, Rbm4, Rc3h1, Stau1, Tia1, Tial1, Ybx1 and Zfp36. In a recent microarray study highlighting transcriptome changes in infected hepatocytes (Albuquerque et al., 2009) we found only the RNA binding protein Fmr1 (fragile X mental retardation 1) and Grb7 (Growth factor receptor-bound protein 7) to be differentially expressed in at least one time-point (out of four studied) during EEF development (6, 12, 18 and 24 hours p.i.). The failure to identify SGs in infected cells corroborates the microarray data and substantiates the finding that *P. berghei* do not induce an SG-defense mechanism in the host liver cell. Equally, only Igf2bp1 (Insulin-like growth factor 2 mRNA-binding protein 1) was identified to be differentially regulated in a study by Chattopadhyay et al. that used HepG2-A16 liver carcinoma cells and *P. falciparum* sporozoites (Chattopadhyay et al., 2011).

In conclusion, our data show for the first time that infection by liver stage malaria parasites does not induce SG formation in the host cell; oxidative and nutritional stress on the other hand lead to the clear formation of SGs in both uninfected and infected HepG2 cells. These data suggest that infected hepatocytes remain competent to respond to strong cellular stressors with global translation repression. It is easy to assume that *Plasmodium* liver stage development, which leads to the production of 10,000 progeny inside the confines of a single hepatocyte with active scavenging of host cell resources, would greatly burden the host cell. Our data suggest that this is likely not the case. The malaria parasite appears to have evolved strategies to ensure that its host cell does not detect either sporozoite invasion or EEF growth as a significant stress requiring a strong global response on the translational level, but rather maintains the host cell in homeostasis.

## Materials and Methods

### *Anopheles stephensi* mosquito maintenance

*Anopheles stephensi* were bred at the insectary of the Instituto de Medicina Molecular. *P. berghei* sporozoites were recovered by hand-dissection from mosquito salivary glands. The *P. berghei* reference line used in this study was GFPcon 259cl2 (Franke-Fayard et al., 2004); it expresses soluble GFP under the control of the *P. berghei* EEF1a promoter throughout the life cycle.

### Hepatoma cell maintenance and infection

The HepG2 human hepatoma cell line was cultured in DMEM supplemented with 10% FCS, 1% penicillin/streptomycin and 1% glutamine, and maintained at 37°C with 5% CO_2_. HepG2 cells were plated on coverslips (50,000 cells per well of a 24-well plate), and infected with 20,000 *P. berghei*-GFP sporozoites per well. Fungizone was added at 1:1000 at the time of sporozoite infection. *P. berghei* parasites expressing soluble GFP were freshly isolated from infected *Anopheles stephensi* mosquitoes for each experiment. SG induction in infected and non-infected cells: 0.5 mM Sodium (meta)arsenite (Sigma) was added to cells for 45 minutes at 37°C to induce SGs, as described (Kedersha and Anderson, 2007).

### Immunofluorescence assay (IFA) and microscopy of SGs

For immunofluorescence analysis (IFA), cells were fixed in 4% PFA for 15 minutes at room temperature (RT), washed 3× in PBS, then permeabilized in ice cold methanol for 10 minutes at −20°C. Cells were washed 5× in PBS, then subsequently blocked in 2% BFA in PBS (BBS) for 30 min, and stained for 2 h at RT with 1° antibodies diluted in BBS. Mouse anti-G3BP (clone 23/G3BP, BD) was used at 1:200, rabbit anti-eIF4G (sc11373, Santa Cruz Biotechnology) was used at 1:200, goat anti-eIF3η (sc16377, Santa Cruz Biotechnology) was used at 1:300, mouse anti-P. berghei HSP70 (2E6) was used at 1:1000, and rabbit anti-GFP directly coupled to AlexaFluor488 (Invitrogen) was used at 1:400. After 5× PBS washes, the cells were incubated with appropriate secondary antibodies and DAPI, for 45 min, washed 3× again with PBS, and mounted in Fluoromount (Southern Biotech). 50 sequential parasites per coverslip and condition were assessed for the presence/absence of SGs in the host cell by microscopy.
